# Bacterial Consortium for Improved Maize (*Zea mays* L.) Production

**DOI:** 10.3390/microorganisms7110519

**Published:** 2019-11-01

**Authors:** Oluwaseyi Samuel Olanrewaju, Olubukola Oluranti Babalola

**Affiliations:** Food Security and Safety Niche Area, Faculty of Natural Science and Agriculture, North-West University, Mmabatho 2735, South Africa

**Keywords:** *Bacillus*, consortia, plant-microbe interactions, principal component analysis, *Pseudomonas*, siderophore production

## Abstract

The ever-increasing human population is a major concern for food security. Maize is the third largest most important food crop. The major problems of cultivation arise from urbanization and land pollution. This reduces the amount of land available for agriculture. The use of chemicals in agriculture is not environmentally friendly. Thus, plant growth-promoting bacteria (PGPB) have been proposed as alternatives. This study aims to test the growth-promoting effect of maize inoculated with six indigenous PGPB isolates. These isolates were assayed for various biochemical and plant growth-promoting activities. They were also assayed for biocontrol activities. Based on the results, six isolates viz A1, A18, A29, NWU4, NWU14, and NWU198 were used to inoculate maize seeds. The inoculated seeds were tried out on the field. A randomized block design was used. PGPB used were in single, consortia of two, and three organisms. The length of the leaves, roots, and stem, plant height, numbers of leaves, and weight of 100 seeds were taken at the fourth and eighth weeks after planting. Microbial consortia increased growth parameters compared to single inoculant treatments. Thus, they can be of advantage in the eradication of low yield. They can also serve as reliable alternatives to chemical fertilizers.

## 1. Introduction

The rhizosphere is the region around the root system. Thus, the soil in this region is the rhizospheric soil. The rhizosphere is in direct contact with the plant root system. This makes it more accessible to nutrients released by the plants. Bacteria found in the rhizosphere are called rhizobacteria. They can either be beneficial or non-beneficial to the plants. The beneficial rhizobacteria are called plant growth-promoting bacteria (PGPB) [1]. Various groups of rhizobacteria, such as Streptomyces, Pseudomonas, Bacillus, Enterobacter, and Azospirillum, are present in the rhizosphere of different crops. Several root-colonizing rhizobacteria strains have, over the years, proven to be good plant growth promoters. The reason behind their usage is to maintain a safe environment. This is achieved through a reduction in using chemical fertilizers in agricultural practices. These chemical fertilizers have detrimental effects on humans and the environment. The plant growth-promoting abilities of PGPB are elicited either directly on the plants or indirectly [2]. Their efficiency in growth promotion has been reported in various plants such as maize [3], rice [4], wheat [5], and others. Their eco-friendly nature gives them an edge over synthetic fertilizers. However, their efficacy on the field and greenhouse is continuously being studied [6,7]. Their mechanisms of action are phytohormone production, ethylene regulation, production of ACC deaminase enzyme, N2 fixation, and phosphate solubilization, siderophore production, hydrogen cyanide (HCN), induce systemic resistance, antibiotics production, synthesis of cell wall degrading enzymes, quorum sensing, and bacteriophages [2].

Their growth-promoting effects vary from one organism to another. This is because of the individual traits they have. The rate at which a rhizobacterium colonizes the rhizosphere determines its shelf life in that environment. The colonizing ability determines its survival rate against other microbes [8]. The type of plant is another key determinant in the rhizobiome make-up. PGPB control pathogens by competing for the available nutrients [9]. Thus, the pathogens might not stand a chance when they are out-numbered.

When it comes to the size of cultivated area and yield, cereals are one of the most important food crops in the world. Among these cereals, maize is of high value [10]. It is one of the most consumed staple food crops in the world. From its use as food to paper and energy industries, as well as its use in livestock feeds [11], we see how valuable it is. Due to its various purposes, its demand is increasing day by day. There is a need to always meet up with the increasing demand. Its nutritional value and high carbohydrate content make it one of the most sought-after food crops [12]. However, its production is hampered by the availability of land, soil type, and rainfall. It is also affected by fall armyworm, maize stalk borer, leaf spot, and leaf blight diseases.

In maize production, PGPB can limit the effect of poor soil through siderophore activities and nitrogen fixation. Phosphate is also very essential for the production of maize. PGPB help to make phosphorus available for the plant. Antibiosis, cyanogenesis, and other biocontrol mechanisms of the indigenous PGPB can control the pests [13,14].

Microbial consortia in agriculture have become an area of interest for growth promotion. Positive interactions between rhizobacteria help in colonization and associative means of combatting pathogens. A single rhizobacterium can have more than one mechanism of action. A combination of two or more PGPB can increase plant growth, combat stress, and control pathogens. To support this claim, studies have shown that the co-inoculation of PGPB improves plant growth [15,16,17,18,19].

The objectives of our study are to test the plant growth-promoting abilities of some indigenous isolates. These isolates will be inoculated as single and consortia organisms. We also want to find out the biocontrol potential of the isolates in the laboratory.

## 2. Materials and Methods

### 2.1. Rhizosphere Soil Sampling

The field site is North-West University farm Mmabatho, Mafikeng, South Africa. Mmabatho municipality’s geographical coordinates are latitude 25°47′S and longitude 25°32′E, having 1281 m altitude. The average annual precipitation is 464 mm and while the average temperature ranges from 6 °C minimum to 30 °C maximum. Agricultural practice is rather intensive. Soil properties of North-West University farm are pH (H_2_O) 5.7, organic carbon 0.69%, particle size distribution (Sand 59.9; silt 31.6; clay 8.5%), and silty loamy sand texture. Soil rhizosphere samples were taken from the Ngaka Modiri Molema District in the North West Province of South Africa. The latitude and longitude of the district are 25°55′N and 25°50′E respectively. It covers a total of 28,206 km^2^. Temperatures range from 17 °C to 31 °C (62 °F to 88 °F) in the summer and from 3 °C to 21 °C (37 °F to 70 °F) in the winter. The average rainfall is 360 mm. Approximately 5–10 g rhizospheric soil was collected from maize plants at a depth of 0–15 cm. During harvest on the maize field, soil samples were collected from the roots of the harvested maize plants. Sterile techniques were employed during each collection. Each sample was labeled immediately and placed in a dry, cool place to prevent moisture accumulation and excessive drying. The samples were then taken to the North-West University’s microbiology research laboratory for analysis.

#### Isolation of Bacteria from Rhizospheric Soils

We suspended 1g of rhizospheric soil in 90 mL of sterile distilled water. The mixture was serially diluted to suitable dilutions (10^−2^, 10^−4^, and 10^−6^). The dilutions were plated in triplicate on Luria–Bertani (LB) agar (Sigma-Aldrich, Saint Louis, MO, USA) for isolation following standard techniques, and plates were incubated at 25 °C for 24 h. Bacteria colonies were sub-cultured and purified by streaking on freshly prepared LB agar plates.

### 2.2. In Vitro Screening of Isolates for Different Plant Growth-Promoting Activities

#### 2.2.1. Phosphate Solubilization

Phosphate solubilization by the isolates was determined by spot inoculation on Pikovskaya agar medium plates containing tri-calcium phosphate (Ca_3_(PO_4_)_2_). After incubation at 28 °C for seven days, the clear zone around the colonies was considered as a positive result for phosphate solubilization activity [20].

#### 2.2.2. Hydrogen Cyanide (HCN) Production

This was carried out according to the method described by Castric [21]. Bacterial cultures were streaked on LB agar medium containing 4.4 g·L^−1^ of glycine. Whatman filter paper No. 1, which was soaked in a solution of 0.5% picric acid in 2% sodium carbonate, was placed inside the lid of the plates, which were then sealed with parafilm and incubated at 30 ± 0.1 °C for four days. The appearance of light brown to dark brown color indicates the positive production of HCN.

#### 2.2.3. Ammonia Production

Production of ammonia (NH_3_) was tested according to the method of Cappuccino and Sherman [22]. A total of 0.5 mL Nessler’s reagent (Water 79.65%, Sodium Hydroxide 12.49%, Mercuric Iodide 4.37%, Potassium Iodide 3.49%) was added to overnight grown cultures which have previously been inoculated in 10 mL peptone broth and incubated at 30 ± 0.1 °C for 48 h in an incubator shaker. NH_3_ production is positive, with the appearance of faint yellow to dark brown color.

#### 2.2.4. Detection of Indole Acetic Acid (IAA)

A total of 500 µL of 24-h old bacterial cultures were used to inoculate 50 mL of LB broth (Sigma-Aldrich), which contains 0.1% (D) l-tryptophan. The product was incubated in a refrigerated incubator shaker at 30 ± 0.1 °C and 180 rpm for 48 h in the dark. The cultures were then centrifuged at a speed of 10,000 rpm for 10 min at a temperature of 4 °C. IAA was estimated in the supernatant using a colorimetric assay [23].

##### Quantification of IAA Production

Five milliliter of DF (Dworkin and Foster, 1958) salts minimal media was used for overnight preparation of isolates. Aliquots of 20 µL from the preparation was then transferred into 5 mL of DF salts minimal media that is supplemented with the following concentrations of l-tryptophan (from a filter-sterilized 2 mg/mL stock prepared in warm water; Sigma): 0, 5, 10, 20, 50, and 100 µg/mL. After 42 h of incubation, a spectrophotometer was used to measure the densities at 530 nm. The bacterial cells were removed from the culture medium by centrifugation (5500× *g*, 10 min). A 1 mL aliquot of the resultant supernatant was mixed vigorously with 4 mL Salkowski’s reagent (150 mL of concentrated H_2_SO_4_, 250 mL of distilled H_2_O, 7.5 mL of 0.5 M FeCl_3_·6H_2_O [23]). The mixture was allowed to stand at room temperature for 20 min before the absorbance was measured at 530 nm. The concentration of IAA in each culture medium was determined by comparison with a standard curve [24].

#### 2.2.5. 1-Aminocyclopropane-1-Carboxylic Acid (ACC) Utilization Assay

This was carried out according to the method of Li et al. [25], which is briefly described. Single colony LB agar was picked into 5 mL of LB broth and incubated at 28 °C overnight on a shaker at a speed of 200 rev min^−1^. Two milliliters of each culture was harvested in a 2 mL micro-centrifuge tube after centrifugation at 8000 g for 5 min. The cell pellets were washed twice using 1 mL of DF medium (Dworkin and Foster, 1958). The washed pellets were then suspended in 2 mL of DF-ACC medium in a 12 mL culture tube and incubated for 24 h at 28 °C on the shaker at a speed of 200 rev min^−1^. A 2 mL sample of DF-ACC medium without inoculation was also incubated. One milliliter of each incubated culture was centrifuged in a 1.5 mL microcentrifuge tube at 8000 *g* for 5 min. One-hundred microliters of the supernatants were diluted to 1 mL using the DF medium in a 1.5 mL microcentrifuge tube.

Sixty microliters of each tenfold diluted supernatant were used for 96-well PCR-plate ninhydrin–ACC assay. DF medium was used as a blank. Each dilution was run in triplicates. The resulted Ruhemann’s Purple color from each dilution and the diluted DF-ACC medium without inoculation was compared by eyes and recorded. After the transfer of 100 µL of the remaining reaction solution, the absorbance of microplate wells was measured at 570 nm using the SpectraMax spectrophotometer (San Jose, CA, USA). An isolate showing a visibly reduced color depth or lower absorbance of supernatant when compared with that of the DF-ACC medium without inoculation was regarded as an ACC-utilizing isolate.

#### 2.2.6. Siderophore Production

##### Detection in Plate Culture

To detect siderophore production, Chrome Azurol S (CAS) agar medium was prepared as described by Schwyn and Neilands [26]. LB agar medium with CAS was inoculated in the plate with 24-h old bacteria and incubated for 72 h at 30 °C. Change in color of the medium from blue to orange or yellow to light orange halo around the colony indicates siderophore production.

##### Quantitative Estimation

It was carried out using a microtiter plate. Supernatants were gotten from 1 mL inoculated broth of each isolate in separate microcentrifuge tubes (Thomas Scientific, Swedesboro, NJ, USA). One-hundred microliters of each supernatant was added in separate wells of microplate followed by the 100 µL CAS reagent. After incubation, optical density was recorded at 630 nm using a microplate reader (Spectra Max M5e, San Jose, CA, USA). Three replicates were taken for each isolate in a 96-well plate, and percentage siderophore was estimated by the formula:

(1) $$\mathrm{Siderophore}\;\mathrm{production}\;[\mathrm{percentage}\;\mathrm{siderophore}\;\mathrm{unit}\;(\mathrm{PSU})]\;=\;\frac{\left(Ar-As\right)*100}{Ar}$$

where *Ar* = absorbance of reference (CAS solution and uninoculated broth), *As* = absorbance of the sample (CAS solution and cell-free supernatant of the sample).

### 2.3. Biochemical Activities of Isolates

#### 2.3.1. Catalase Production

A drop of 30% hydrogen peroxide was added to a 24 h old bacterial colony on a clean glass slide and mixed with a sterile toothpick. The subsequent effervescence shows catalase activity [27].

#### 2.3.2. Protease Production

Assay for protease production was done according to the method of Maurhofer et al. [28]. Each bacterial isolate was spotted on a skim milk agar plate and incubated for 24 h. The isolate showing a halo zone around its colony was considered positive for protease production.

#### 2.3.3. Oxidase Production

Oxidase production by the isolates was carried out using a filter paper spot method [29]. Kovács oxidase reagent (1–2 drops) was added to 24-h old culture on a small piece of filter paper resulting in a change in color to dark purple within 60 to 90 s, which indicates positive for oxidase production.

### 2.4. Antagonism Assay Against Phytopathogenic Fungi

In vitro bacterial antifungal activities against *Fusarium graminearum* was done by the slight modification using the method of Gopalakrishnan et al. [30] on Potato Dextrose Agar (PDA) medium. The isolates were streaked 3 cm in the distance opposite pathogenic fungi inoculated at the center of the medium. The barrier between isolates and fungi indicated an antagonistic interaction between them. The antagonistic activity was investigated for four to seven days after incubation at 25 °C.

### 2.5. Molecular Characterization of Bacterial Strains

Isolates that showed the best plant growth-promoting traits were picked for further characterization and identification. The *Streptomyces* isolates, designated as NWU4, NWU14, and NWU198 used in this study, have previously been identified [31].

#### 2.5.1. DNA Extraction and 16S rDNA Gene Amplification

Genomic DNA of all selected isolates was extracted using ZR soil Microbe DNA MiniPrepTM (Zymo Research, Irvine, CA, USA) extraction kit according to the manufacturer’s instructions. PCR was carried out following standard protocols. The 16S rDNA genes were amplified using universal bacterial primers 27f and 1525r (Forward AGAGTTTGATCMTGGCTCAG, Reverse AAGGAGGTGWTCCARCC). PCR was performed using a DNA Engine DYADTM Peltier thermal cycler (Bio-Rad, Hercules, CA, USA). The PCR protocol used was as follows; initial denaturation at 95 °C for 5 min, followed by 35 cycles of denaturation at 94 °C for 30 s, annealing was done at 62 °C for 30 s with an extension at 72 °C for 1 min, and finally another extension at 72 °C for 5 min.

#### 2.5.2. Sequence Alignment and Phylogenetic Analysis

Sequence alignment was performed with the Chromas software version 2.6.6.0 (Technelysium Pty. Ltd., South Brisbane QLD, Australia) and Bioedit version 7.2.5.0. The phylogenetic tree was constructed using the MEGA software version 7.0.14 Phylogenetic analysis of the 16S rDNA sequences was performed by the maximum parsimony method.

### 2.6. Preparation of Inoculum and Seed Treatment for Field Trial

#### 2.6.1. Preparation of Inoculum and Seed Treatment

Isolates used as inoculum were selected based on the complementarity of their respective activities in the in vitro tests carried out (Table 1 and Table 2). Bacterial isolates were grown for 24 h in LB broth (Sigma-Aldrich, Saint Louis, MO, USA), and serial dilution was done to the level of 10^5^ with sterilized distilled water. Maize seeds to be used for planting were surface sterilized with mercury chloride for 5 min and rinsed five times with sterilized distilled water. Inside a 100 mL falcon tube, seeds were inoculated with 1 µL of 10^5^ colony-forming unit (CFU) mL^−1^ bacterial dilution. Bacterial inoculants were in single, double, or triple treatments. After inoculation, they were left for four to six hours with occasional shaking. For controlled treatment, seeds were soaked in sterile distilled water.

#### 2.6.2. Field Study

Field trials were carried out (January to March 2017) using the selected isolates and the *Streptomyces* sp. strains mentioned earlier. The *Streptomyces* sp. strains were included to test their activities on plant growth promotion as most studies on them are majorly on biocontrol abilities through the production of secondary metabolites [32]. All isolates used for field trials were selected based on their in vitro activities. Phosphate solubilization is key to plant growth because of the function of phosphate in metabolism. In addition to the ability to be able to solubilize phosphate, the three isolates that strong biochemical with strong plant growth-promoting attributes were selected because some isolates have strong biochemical attributes but weak plant growth promoting attributes and vice versa. The field plot microbiota was similar across all treatments as it was not depleted before planting. The design employed was a randomized complete block design (RCBD) with three replicates. Data on plant height, number of leaves, length of root, and length of the stem were recorded at four weeks and eight weeks after planting.

The treatment combinations used are: Single treatments—A1, A18, A29, NWU4, NWU14, NWU198; Double treatments—A (1 + 18), A (1 + 29), A1+NWU19; Triple treatments—A (1 + 18 + 29), NWU (4 + 14+ 198); The control was treated with sterilized distilled water only.

### 2.7. Statistical Analysis

Data from each growth parameters were transformed into log_10_ to improve the homogeneity of the variance before ANOVA was performed on the transformed data to find out whether there is any difference between groups on specific treatments and the degree of significance of the differences among the variables was determined using mean values considering the standard deviation (*n* = 3). Anywhere F value is significant; posthoc test was carried by Duncan’s Multiple Range Test [33] at 5% probability. The analysis was carried out using the software Statistical Analysis computer package (SAS) version 9.4 (SAS Institute, Inc., Cary, NC, USA), and the graphs were plotted using GraphPad Prism 7 (https://www.graphpad.com/). Principal component and cluster analysis were performed to compare the means of the treatments with growth parameters evaluated.

## 3. Results

### 3.1. In Vitro Screening of Isolates for Plant Growth-Promoting Activities

In the present study, 31 isolates were isolated and characterized according to their plant growth promotion assays (Table 1). These isolates were labeled A1 to A31 and three Streptomyces isolates from a previous study of Adegboye and Babalola [31] were also included.

#### 3.1.1. IAA Production and ACC Utilization Assay

**Figure 1:**
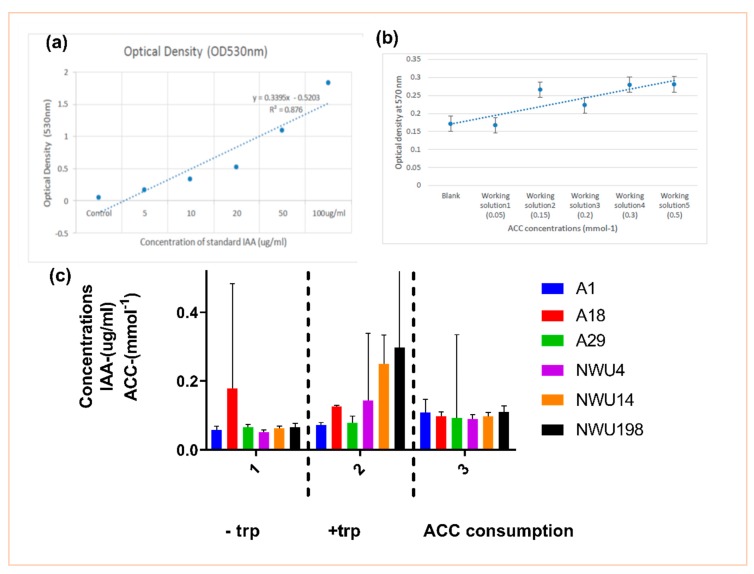
Indole acetic acid (IAA) and 1-aminocyclopropane-1-carboxylic acid (ACC) production by isolates. (**a**) Standard graph of IAA at an optical density of 530 nm. (**b**) Standard curve of ACC concentrations ranging from 0.05 to 0.5 mmol^−1^ determined by the 96-well polymerase chain reaction (PCR)-plate ninhydrin assay (*y* = 0.0242*x* + 0.1467, *R*² = 0.7364). Each data point represents the mean of triplicate determinations, and the error bar represents the standard error. (**c**) IAA production in the absence and presence of tryptophan (trp), and ACC consumption level of the isolates. No significant difference was observed after running the ANOVA analysis.

The production of IAA in this study was carried out both in the presence and absence of tryptophan (Figure 1). Like most studies, more IAA was synthesized in the presence of tryptophan. More than 80% of PGPB produce IAA, and its effect on plant growth is directly proportional to its concentration [34]. IAA also helps to withstand drought stress through root elongation. 

The level of ACC consumption increases in the *Streptomyces* isolates NWU 14 and NWU 198 followed by the *Bacillus subtilis* A1. The *Pseudomonas* spp. happen to show minimal consumption of ACC (Figure 1).

#### 3.1.2. Siderophore Production

**Figure 2:**
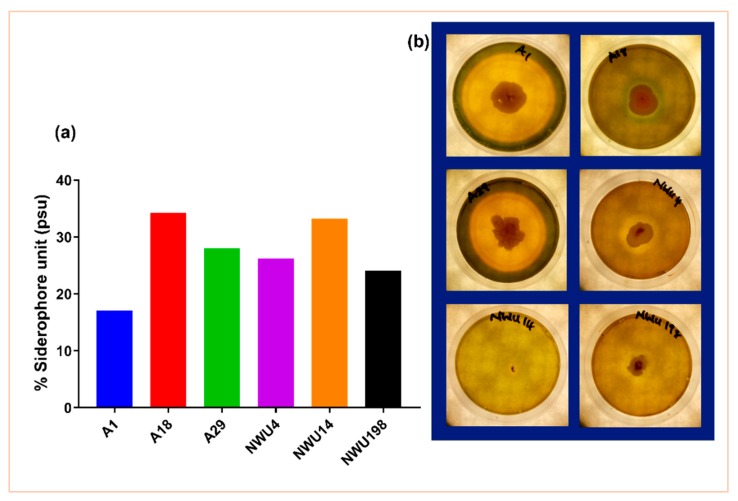
Siderophore production by isolates. (**a**) Mean amount of percentage siderophore unit production in each rhizobacterium. (**b**) Halo zones are seen in siderophore-producing rhizobacteria.

High production of siderophore was seen in isolate A1 (Figure 2). Siderophore correlates with a significant nutrient acquisition by plants and indirect pathogen suppression through nutrient deprivation [2].

### 3.2. Biochemical Activities of Isolates

The 31 isolates responded to the different biochemical tests in different ways (Table 2).

From the results obtained in Table 1 and Table 2, three isolates were selected. The selection was based on their ability to solubilize phosphate, and other reasons are given earlier. Phosphate is present in the soil in the form that is not accessible to plants. Plants need it for energy to drive various metabolic processes. Thus, the ability to solubilize phosphate for plant use is one of the major traits of a good PGPB. Among the tested isolates, *Pseudomonas* sp. A18 was the best phosphate solubilizer, while *Bacillus subtilis* A1 and *Pseudomonas* sp. A29 were slight phosphate solubilizers. They also complemented their phosphate activity with good biochemical activities. These prompted their selection for antagonism assay molecular identification, and field study.

### 3.3. Antagonism Assay Against Phytopathogenic Fungi

**Figure 3:**
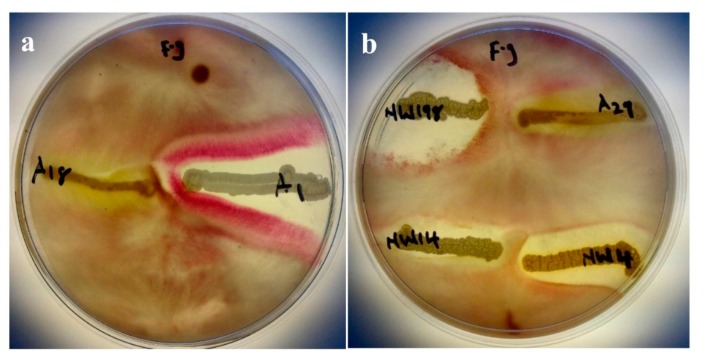
Antifungal activity against *F. graminearum* (*F*. *g*.). In vitro assay of the six selected isolates on *F. graminearum*. plate (**a**) shows activities of isolates A18 and A1 while (**b**) shows that of isolates NW198, A29, NW14, and NW4 against *Fusarium graminearum.*

Four out of the six isolates showed strong antagonistic activity against *Fusarium graminearum*. Only isolates A18 and A29 did not show any inhibitory effect (Figure 3). Isolates A1 and NWU198 showed higher inhibition effect in the reduction of pathogen growth. They were followed by NWU14 and NWU4, both showing a considerable inhibitory effect. Isolate A18 did not inhibit the growth of *Fusarium graminearum* and it did not produce any protease. Others were all positive inhibitors, but not all were able to produce protease enzyme. HCN was produced by only A18 and NWU4.

### 3.4. Molecular Characterization of Bacterial Strains

**Figure 4:**
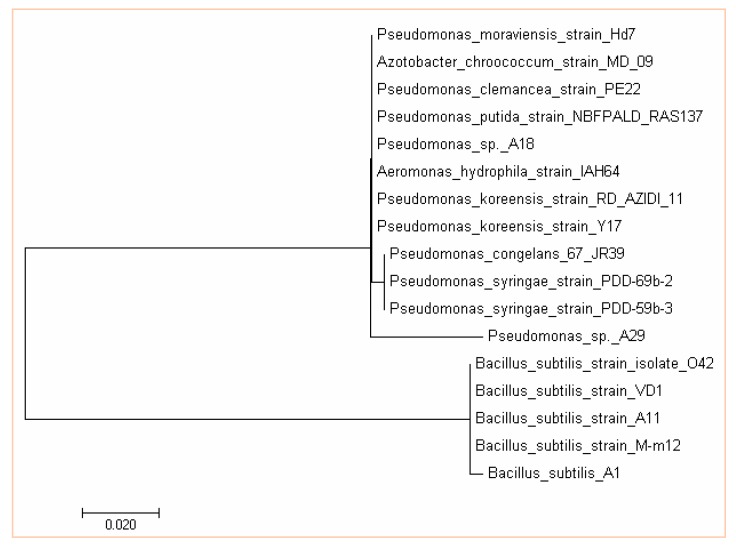
Evolutionary relationships of taxa of *Bacillus subtilis* and *Pseudomonas* sp. The evolutionary history was inferred using the maximum parsimony method. The bootstrap consensus tree inferred from 1000 replicates is taken to represent the evolutionary history of the taxa analyzed. The evolutionary analysis was conducted in MEGA7.

Consistent with this considerable phenotype heterogeneity, comparative gene sequencing clearly showed diversity in the genera. The phylogenetic relationship between the isolates was determined by 16S rDNA sequence analysis (Figure 4). The partial nucleotide sequences of the 16S rDNA gene of the isolates were compared with the nucleotide database of NCBI (National Center for Biotechnology Information, Bethesda, MD, USA) web server through the basic local alignment search tool (BLAST) (Bethesda, MD, USA).

Three strains of *Streptomyces* which have already been identified in the works of Adegboye and Babalola [31] were also used. A18 and A29 were identified as *Pseudomonas* sp., while A1 was identified as *Bacillus subtilis* (Figure 4). Accession numbers KX453173 to KX453175 which have been deposited in the GenBank, have been assigned to isolates A1, A18 and A29. NWU 4, NWU 14 and NWU 198 are *Streptomyces globisporus*, *Streptomyces griseoflavus,* and *Streptomyces heliomycini* which have previously been identified by Adegboye and Babalola [31] as reported earlier.

### 3.5. Plant Growth Promotion Assay

For the parameters taken, no high differences were observed. The major difference of note is that of the control compared to others.

At four weeks, the *Pseudomonas* sp. A29-treated plants have the longest leaf length, while at eight weeks, the consortia, A (1+18+29) have the longest leaf length (Table 3). But, the *Pseudomonas* sp. A29-treated plant seems to somehow retrogress in leaf length at the 8th week. Like the leaf length at four weeks, the A29-treated plants show the greatest effect on root length along with the triplicate treatments. But at 8 weeks, both the triplicate and double isolate-treated plants of A (1 + 29) showed an increase in the observed root length of the maize plants (Table 3). In both fourth and eighth weeks, the plants treated with two organisms, A (1 + 18) and A (1 + 29), have increased stem length compared to the other treatments. In this regard, plants treated with A (1 + 8 + 29) also showed an increase. The number of leaves was greater with the A (1 + 18 + 29) consortium at four weeks, while at eight weeks, A (1 + 18) had the highest number of leaves. Compared to control and single treatments, consortium generally had the highest number of leaves. Isolated A29-treated plants showed an increase in plant height at four weeks. However, at eight weeks, consortia of “A1+NWU198” and A (1 + 18 + 29) have the highest values. However, in the HSWT, consortium showed more effect than the other treatments.

## 4. Discussion

Due to the importance of phosphate in metabolic processes as an energy carrier, the ability of the isolates to solubilize phosphate was considered as a criterion along with other biochemical results for the selection of the isolates used in this study. Those with strong plant growth-promoting abilities and complementing biochemical activities were selected. The *Streptomyces* species used were selected randomly from a past study. The study from which the *Streptomyces* isolates were selected was based on the characterization of the actinomycetes present in the soil by Adegboye and Babalola [31]. The soil was taken from the same geographical location as that of this study.

The use of PGPB in plant growth promotion and disease control has been reported in many studies [3,13]. Different mechanisms are employed by these PGPB in carrying out these functions. Inoculating plants with bacteria for plant growth promotion is gradually becoming acceptable. In the soil, these microbes all thrive and live together based on their compatibility and ability to survive. These attributes led researchers to the idea of co-inoculation. The use of consortia organisms in plant growth promotion has been evaluated on various crops [15,35].

Only 23.5% of the isolates used in this study were able to solubilize phosphate. This is not up to half of the total number of isolates. Bearing in mind the importance of phosphate in metabolic processes, this trait is important in PGPB activities [36]. In like manner, 14.7% produced HCN. HCN production is one of the mechanisms used in combatting pathogens [37]. It is a major biocontrol attribute for crop protection. This is commonly produced by *Pseudomonas* [38]. It is a phytotoxic agent that inhibits the enzymes of major metabolic pathways. Thus, the increased interest in its application as biocontrol agents [34]. 35.5% of the total isolates in the initial screening produced IAA. IAA functions in root elongation. Its synergy with ethylene helps in the plant’s defense system [39]. Further screening of the six selected isolates showed some discrepancies in the production of IAA. The assay was done with media amended with tryptophan and the one not amended (Figure 1). Tryptophan is the precursor in IAA synthesis. Thus, the availability of tryptophan increases the amount of IAA. IAA is important for cell growth and division [40]. The result in this study is consistent with what reports of Kumawat et al. [41] in the endophytic bacteria of Soybean. Activities of the ACC deaminase enzyme helps in regulating the production of ethylene. This, in turn, promotes stress alleviation [42]. Iron is the fourth abundant element, and it is highly important in plant growth and reproduction. Iron has an important role in the light-absorbing pigment chlorophyll for photosynthesis. Siderophore-producing PGPB prevents pathogens from getting access to Fe^3+^ in the rhizosphere. They bind to the available Fe^3+^ [2] and make it accessible to plants. In this study, the result of the quantitative siderophore production does not correspond with that of the plate assay. The reason for this is not well understood. Although we can deduce that they give different views; this might be a result of differences in experimental procedures and the regimes in the various procedures. The type of siderophore produced might also play an important role in this regard. This may invariably depend on the amount and accessibility of nutrients [43]. Furthermore, we observed that siderophore production does not tally with the antifungal assay. *Bacillus subtilis* A1 and *Streptomyces heliomycini* 198 produce the lowest amount of percentage siderophore. But they both have the highest on the pathogen. Isolate A1 has the biggest halo zone in the plate assay. Isolate A18 is arguably the only isolate that conforms with both assays as it gives the same outcome in both. Isolate A18 being a *Pseudomonas* species supports various studies about *Pseudomonas* being siderophore producers. Findings of the effect of siderophore on biocontrol abilities were reported in the studies of Arya et al. [44], Verma et al. [45], and Tortora et al. [46] among others. The ability of the *Bacillus* and *Pseudomonas* genus to produce siderophore is further supported in the reports of Rajendran et al. [47]. Through the use of siderophore, *Bacillus subtilis* strains have been shown to suppress pathogens in various plants [35,48,49].

Catalase, oxidase, and protease represent some of the hydrolytic enzymes used by rhizobacteria. They act by hydrolyzing cell walls of invading pathogens as well as destroy their oospores [2]. Catalase, oxidase, and protease were produced by 94, 56, and 32.4% of the total isolates in this study, respectively. Enzyme production in PGPB was successfully used in consortium with other biocontrol agents Someya et al. [50] and Thakkar and Saraf [51].

*Fusarium graminearum* is a major cereal fungal pathogen. Reports have shown that it affects maize crops [52,53,54]. Five of the selected six strains showed antagonistic activity against the pathogen in vitro (Figure 3). This antagonism can be related to their ability to produce secondary metabolites in the form of siderophores, HCN, and some biochemical enzymes (Table 1 and Table 2, Figure 3). *Streptomyces* have been immensely used in this regard as they are good producers of secondary metabolites [31,32]. *Bacillus subtilis* are also known biocontrol agents, as indicated in the study by Mousa et al. [55]. In their study, they isolated some bacterial endophytes and tested them against *Fusarium graminearum*. *Bacillus subtilis* QST713 which is a commercial biocontrol agent, was the control. *Bacillus subtilis* strain SG6 was also used against *Fusarium graminearum* in the study conducted by Zhao et al. [56]. They got a high percentage of inhibition from this strain on the pathogen. The result of this study is in tandem with the two studies mentioned. The inability of *Pseudomonas* sp. A18 to inhibit the growth of *Fusarium graminearum* in this study is not known but *Pseudomonas* sp. A29 was able to effect some level of inhibition. We can say that not all *Pseudomonas* sp. used can be effective biocontrol agents against the tested pathogen. The ability to fully identify the isolates to the species level would have been valuable. We would have been able to easily identify the two species and know the one that can inhibit and the one that cannot. *Streptomyces* species in this study were effective in inhibiting *Fusarium graminearum*. This result agrees with the studies of Bressan and Figueiredo [57] on maize plants. Vijayabharathi et al. [58] also showed the biocontrol ability of *Streptomyces* sp. in chickpea. Such biocontrol activities can be attributed to the production of wall degrading enzymes, siderophore, HCN, and antibiotics [14,32].

Comparative gene sequencing clearly showed diversities in the genera. Isolate A1 was found to be closely related to *Bacillus subtilis* strain M-m12, while isolate A18 is closely related to *Pseudomonas putida* strain NBFPALD-RAS137, and isolate A29 is found to be closely related to *Pseudomonas congelanse* 67-JR39. Previous findings showed that similar organisms to the isolates also possess plant growth-promoting activities [9]. Identified *Streptomyces* genera are also not left out in plant growth promotion majoring as biocontrol agents [59].

Together, the present findings give a mixed notion about the plant growth-promoting abilities of the consortium of rhizobacteria with respect to single isolates. Interactions between isolates can either be positive or negative. This will have an effect on the activities of the consortia inoculants. A majority of them performed better in all parameters than the single treatments and control (Table 3). All parameters were found to be significantly different in both the 4th and 8th weeks. A (1 + 18 + 29) did not show any significant difference in the 4th and 8th weeks. At four weeks after planting, maximum root length, stem length, and plant height were recorded by the single treatments. At eight weeks after planting, all parameters were maximum with the consortium treatments. The only exception was the number of leaves which is the only one that has a maximum with a single treatment. PGPB is able to release molecules that can directly or indirectly increase plant growth [2,13,60,61]. LOL was also not significantly different in the 4th and 8th weeks after planting. A single inoculation of A29 and consortium A (1 + 18) also did not show any significant difference in the LOS parameter. Although there were some significant growth responses between treatments. Consortium majorly performing better than the single inoculants. This result is supported by the work of Vijayabharathi, Gopalakrishnan, Sathya, Vasanth Kumar, Srinivas, and Mamta [58] who reported that *Streptomyces* consortium increased chickpea growth. Argaw [62] also reported an improved yield with *Bradyrhizobium japonicum* and *Pseudomonas* sp. co-inoculation on Soybean. Likewise, Kumar, Pandey, Dubeya, and Maheshwari [15] reported improved nutrient uptake, nodulation, and growth of common bean. Furthermore, an increase in root length due to inoculation with bacterial consortium was also reported in the study of Akhtar et al. [63]. HSWT was greater in consortium treated plants than other treatments. This is due to the ability to solubilize phosphate, produce siderophore, IAA, and ACC deaminase. These help in nutrient uptake, root elongation, stress relieve, and antagonistic activities for rhizosphere competition by PGPB [2].

**Figure 5:**
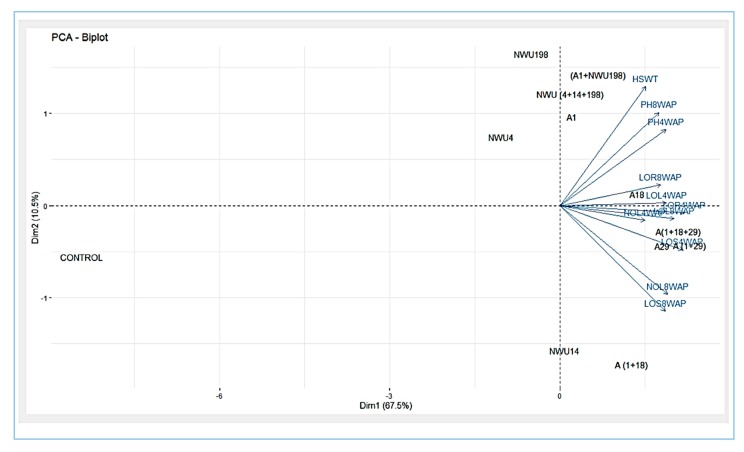
Principal component analysis of growth traits in maize.

**Figure 6:**
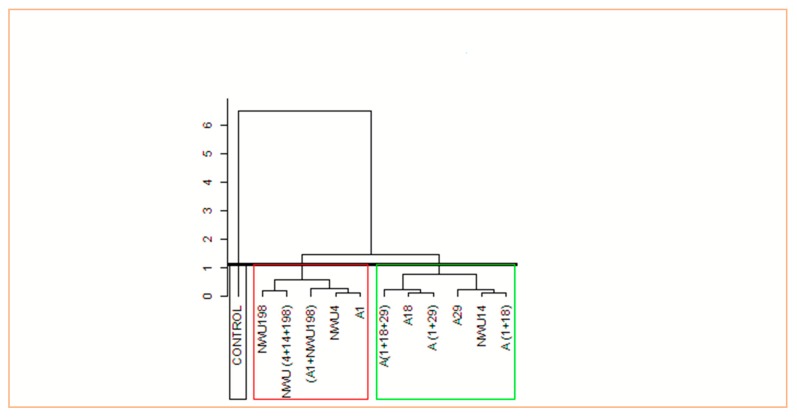
Hierarchical clustering of the treatments on growth parameters.

Principal Component Analysis (PCA) was used to investigate the relationship between the applied treatments and growth parameters. Principal component 1 (PC1), which relates to dim 1 and principal component 2 (PC2), which relates to dim 2 in the biplot, accounted for 67.5% and 10.5% of the variations, respectively (Figure 5). In PC1, (A1 + NWU198), NWU (4 + 14 + 198), A1, A18, A (1 + 18 + 29), A29, A (1 + 18), and A (1 + 29) all had significant influences on all the growth parameters evaluated. (A1+NWU198), NWU (4+14+198), A1, and A18 have a significant impact on HSWT, PH, root length, and leaf length at four weeks after planting while A (1 + 18 + 29), A29, A (1+29), A (1 +18), and NWU14 have a significant impact on the number of leaves, length of the stem, and leaf length at 8 weeks after planting. Cluster analysis divided the treatments into three categories based on their impact on the growth parameters (Figure 6, Supplementary Figure S1).

A1-Bacillus subtilis, A18-*Pseudomonas* sp., A29-*Pseudomonas* sp., NWU4-*Streptomyces globisporus*, NWU14-Streptomyces griseoflavus, and NWU198-*Streptomyces heliomycini*.

The field provides a good platform to test the ability of these isolates in a natural environment. All isolates can effectively be used for sustainable maize crop production. But in virtually all treatments, the triple treatment proves to be more effective. It is very important to know that not all combinations gave more increase than single treatments, and not all triple combinations happen to be more effective. Some triple applications were less-effective compared to others. Researches on PGPB should focus on the possible interactions between isolates. This can help to select the best combinations for good bioinoculant formulation.

In conclusion, our results indicated that isolates A1, A18, A29, NWU4, NWU14, and NWU198 and their consortium enhances Maize growth. They will be beneficial in Maize cultivation. Although the biocontrol abilities of these isolates were not tested on the field; however, the in vitro result showed that they have the potential to be good biocontrol agents against *Fusarium graminearum*. Further works on colonization ability and potential interactions between isolates will increase the efficiency of PGPB. Proper elucidation of interactions in consortium inoculants will enhance acclimatization in the rhizosphere.

## Figures and Tables

**Table 1:** Plant growth-promoting properties of bacterial isolates.

Bacterial Isolates	Ammonia Production	HCN Production	IAA Production	Phosphate Solubilization
A1, A4, A6	++	−	−	+
A2, NWU 4	+	+	−	−
A3	+	+	−	+
A5, A7, A9, A10, A13, A16, A19, A21, A25, A28, A30, NWU 14, NWU 198	+	−	−	−
A8	−	−	−	−
A11, A23	++	−	+	−
A12, A14	+	−	+	−
A15, A17	++	−	+	+
A18	++	+	+	++
A20	++	++	+	++
A22, A24, A27, A31	++	−	−	−
A26	−	++	+	++
A29	++	−	+	+

+, production; ++ significant production; −, no production.

**Table 2:** Biochemical characterization of isolates.

Bacterial Isolates	Catalase Production	Oxidase Production	Protease Production
A1, A2	+	+	+
A3, A18	+	++	−
A4, NWU14, NWU198	++	+	−
A5, A6, A10, A24, NWU4	+	+	−
A7, A9, A16, A25	+	−	+
A8, A26	−	−	−
A11, A23	+	++	+
A12, A14, A15, A19, A31	+	−	−
A13, A17, A20, A21	++	−	−
A22, A29	++	++	−
A27, A28	++	+	+
A30	++	++	+

+, slight production; ++, significant production; −, no production.

**Table 3:** Growth related traits at 4 ^th^ and 8 ^th^ weeks after planting.

Treatments	LOR4WAP (cm)	LOR8WAP (cm)	LOL4WAP (cm)	LOL8WAP (cm)	LOS4WAP (cm)	LOS8WAP (cm)	NOL4W AP	NOL8W AP	PH4WAP (cm)	PH8WAP (cm)	HSWT (g)
Control	0.84 ± 0.06 ^e^	1.08 ± 0.06 ^f^	0.97 ± 0.01 ^c^	1.16 ± 0.04 ^b^	1.53 ± 0.01 ^d^	1.62 ± 0.03 ^e^	0.92 ± 0.03 ^d^	0.95 ± 0 ^b^	1.63 ± 0.05 ^d^	1.86 ± 0.04 ^c^	1.35 ± 0.02^d^
A1	1.14 ± 0.05 ^bcd^	1.22 ± 0.03 ^ed^	1.31 ± 0.02 ^ab^	1.34 ± 0.02 ^a^	1.74 ± 0.03 ^abc^	1.77 ± 0.07 ^cd^	0.98 ± 0.03 ^abcd^	1.01 ± 0.05^ab^	1.87 ± 0.12 ^abc^	2.04 ± 0.03 ^ab^	1.47 ± 0.03 ^abc^
A18	1.2 ± 0.03 ^ab^	1.27 ± 0.02 ^bc^	1.31 ± 0.04 ^b^	1.37 ± 0.04 ^a^	1.69 ± 0.03 ^abc^	1.78 ± 0.02 ^bc^	1.02 ± 0.06 ^abc^	1.05 ± 0.02 ^ab^	1.94 ± 0.01 ^a^	2.03 ± 0.02 ^ab^	1.44 ± 0.04 ^bc^
A29	1.23 ± 0.01 ^a^	1.26 ± 0.02 ^cd^	1.34 ± 0.04 ^a^	1.37 ± 0.06 ^a^	1.79 ± 0.03 ^ab^	1.77 ± 0.02 ^bc^	0.97 ± 0.06 ^abcd^	1.09 ± 0.04 ^a^	1.88 ± 0.04 ^abc^	2.04 ± 0.03 ^ab^	1.45 ± 0.05 ^bc^
NWU4	1.10 ± 0.04 ^d^	1.19 ± 0.01 ^e^	1.28 ± 0.06 ^ab^	1.34 ± 0.05 ^a^	1.73 ± 0.06 ^abc^	1.73 ± 0.04 ^cd^	0.95 ± 0.10 ^bcd^	1.01 ± 0.02 ^ab^	1.87 ± 0.01 ^abc^	2.01 ± 0.072 ^ab^	1.46 ± 0.04 ^bc^
NWU14	1.15 ± 0.02 ^abcd^	1.27 ± 0.02 ^bcd^	1.31 ± 0.09 ^ab^	1.43 ± 0.06 ^a^	1.74 ± 0.02 ^abc^	1.76 ± 0.05 ^ab^	0.98 ± 0.03 ^abcd^	1.05 ± 0.08 ^ab^	1.81 ± 0.01 ^c^	1.98 ± 0.02 ^b^	1.42 ± 0.03 ^c^
NWU198	1.12 ± 0.02 ^cd^	1.31 ± 0.02 ^ab^	1.24 ± 0.08 ^b^	1.28 ± 0.06 ^a^	1.69 ± 0.04 ^c^	1.76 ± 0.02 ^d^	0.97 ± 0.03 ^abcd^	1.01 ± 0.05 ^ab^	1.88 ± 0.05 ^abc^	2.01 ± 0.05 ^ab^	1.49 ± 0.02^ab^
A (1 + 18)	1.19 ± 0.04 ^abc^	1.26 ± 0.03 ^bcd^	1.36 ± 0.01 ^b^	1.46 ± 0.04 ^a^	1.71 ± 0.06 ^a^	1.76 ± 0.01 ^a^	0.97 ± 0.07 ^abcd^	1.09 ± 0.02 ^a^	1.88 ± 0.03 ^abc^	1.99 ± 0.05 ^b^	1.44 ± 0.03 ^bc^
A (1 + 29)	1.2 ± 0.05 ^abc^	1.34 ± 0.04 ^a^	1.35 ± 0.01 ^ab^	1.44 ± 0.03 ^a^	1.74 ± 0.01 ^a^	1.75 ± 0.01 ^ab^	1.04 ± 0.04 ^ab^	1.06 ± 0.06 ^a^	1.90 ± 0.04 ^abc^	2.02 ± 0.02 ^ab^	1.47 ± 0.03 ^abc^
(A1 + NWU198)	1.15 ± 0.01 ^abcd^	1.25 ± 0.01 ^cd^	1.29 ± 0.06 ^b^	1.34 ± 0.06 ^a^	1.72 ± 0.05 ^abc^	1.72 ± 0.07 ^cd^	1.01 ± 0.02 ^abcd^	1.04 ± 0.04 ^ab^	1.92 ± 0.02 ^ab^	2.08 ± 0.04 ^a^	1.47 ± 0.01 ^abc^
A (1 + 18 + 29)	1.22 ± 0.02 ^a^	1.34 ± 0.016 ^a^	1.32 ± 0.02 ^b^	1.43 ± 0.01 ^a^	1.70 ± 0.03 ^abc^	1.79 ± 0.02 ^ab^	1.05 ± 0.02 ^a^	1.05 ± 0.08 ^ab^	1.83 ± 0.07 ^bc^	2.01 ± 0.05^ab^	1.50 ± 0.02^ab^
NWU (4 + 14 + 198)	1.2 ± 0.07 ^abc^	1.33 ± 0.03 ^a^	1.25 ± 0.04 ^b^	1.36 ± 0.04 ^a^	1.71 ± 0.03 ^bc^	1.72 ± 0.01 ^bcd^	0.94 ± 0.03 ^cd^	1.04 ± 0.08 ^ab^	1.88 ± 0.02 ^abc^	1.99 ± 0.02 ^b^	1.52 ± 0.01 ^a^

Numbers representing means ± standard deviation in a column followed by the same letter are not significantly different according to Duncan’s multiple range tests (*p* ≤ 0.05). Length of leaves = LOL, length of root = LOR, length of stem = LOS, plant height = PH, number of leaves = NOL, and weight of 100 seeds = HSWT. WAP = weeks after planting.

## Supplementary Materials

The following are available online at https://www.mdpi.com/2076-2607/7/11/519/s1, Figure S1: Factor map showing the clustering of the treatments into 3 groups.
