# Supplementary figures and images for: Bacterial Consortium for Improved Maize (Zea mays L.) Production
Source: Microorganisms. 2019 Nov 1;7(11):519. doi: 10.3390/microorganisms7110519 (PMC6920993; doi:10.3390/microorganisms7110519)

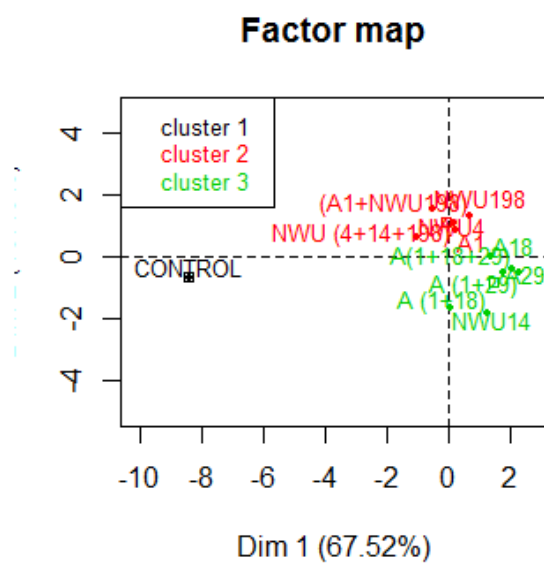

**Figure S1.** Factor map showing the clustering of the treatments into 3 groups.

Supplement: Supplementary file 1 [file microorganisms-07-00519-s001.pdf]
